# Turnkey algorithmic approach for the evaluation of gastroesophageal reflux disease after bariatric surgery

**DOI:** 10.1093/gastro/goad028

**Published:** 2023-06-09

**Authors:** Omar M Ghanem, Rabih Ghazi, Farah Abdul Razzak, Fateh Bazerbachi, Karthik Ravi, Leena Khaitan, Shanu N Kothari, Barham K Abu Dayyeh

**Affiliations:** Department of Surgery, Mayo Clinic, Rochester, MN, USA; Department of Medicine, Mayo Clinic, Rochester, MN, USA; Department of Medicine, Mayo Clinic, Rochester, MN, USA; CentraCare, Interventional Endoscopy Program, St Cloud Hospital, St Cloud, MN, USA; Department of Medicine, Mayo Clinic, Rochester, MN, USA; Department of Surgery, Case Western Reserve University, Cleveland, OH, USA; Department of Surgery, Prisma Health, Greenville, SC, USA; Department of Medicine, Mayo Clinic, Rochester, MN, USA

**Keywords:** RYGB, sleeve gastrectomy, bariatric surgery, gastroesophageal reflux disease, GERD

## Abstract

Bariatric surgeries are often complicated by de-novo gastroesophageal reflux disease (GERD) or worsening of pre-existing GERD. The growing rates of obesity and bariatric surgeries worldwide are paralleled by an increase in the number of patients requiring post-surgical GERD evaluation. However, there is currently no standardized approach for the assessment of GERD in these patients. In this review, we delineate the relationship between GERD and the most common bariatric surgeries: sleeve gastrectomy (SG) and Roux-en-Y gastric bypass (RYGB), with a focus on pathophysiology, objective assessment, and underlying anatomical and motility disturbances. We suggest a stepwise algorithm to help diagnose GERD after SG and RYGB, determine the underlying cause, and guide the management and treatment.

## Introduction

The modern global spread of obesity has reached pandemic heights. According to the World Health Organization, obesity has nearly tripled since 1975, affecting 650 million individuals in 2016 [1]. Bariatric surgery is the most effective obesity treatment, with 256,000 bariatric procedures performed in the USA in 2019. Currently, the most frequently performed bariatric surgery is sleeve gastrectomy (SG) (61.4%), followed by Roux-en-Y gastric bypass (RYGB) (20.8%) [2].

Gastroesophageal reflux disease (GERD) is a common obesity co-morbidity, with an incidence ranging from 22% to 70% [3]. The underlying pathophysiology of GERD in this patient population is multifactorial, but it is mainly related to lower esophageal sphincter (LES) and hiatal crural muscle injury, which are essential components of the anti-reflux barrier. A study on patients being evaluated for reflux showed an increased prevalence of mechanically defective LES in patients with higher body mass index, especially in those with a concomitant hiatal hernia [4]. Other contributing factors include increased intra-abdominal and intragastric pressures, decreased length of the intra-abdominal portion of the LES, and abnormal esophageal peristalsis [5–9].

The relationship between GERD, obesity, and bariatric surgery is intertwined. Weight loss itself is associated with an alleviation of GERD symptoms [10, 11]. In fact, RYGB is an appropriate treatment for patients with obesity and GERD [12]. Similarly, a proportion of patients may experience improvement in GERD symptoms after SG [13]. On the other hand, GERD is a well-known complication of bariatric surgery, especially SG, with a significant proportion of patients experiencing worsening of pre-existing GERD or developing de-novo GERD [14, 15].

Several studies have evaluated GERD objectively in this patient population and reported significant rates of erosive esophagitis and Barrett’s esophagus, as well as abnormal acid and non-acid esophageal exposure on pH studies. Similarly, esophageal dysmotility and anatomical abnormalities have been documented, and are thought to be involved in the pathophysiology of GERD after bariatric surgery.

The growing rate of obesity worldwide is expected to yield a simultaneous increase in bariatric surgeries, and consequently an increase in the number of patients requiring evaluation of GERD after surgery. A thorough post-surgical evaluation in a patient with new-onset or refractory reflux is warranted to improve the patient’s quality of life and mitigate the sequelae of GERD, including erosive esophagitis, Barrett’s esophagus, and esophageal adenocarcinoma [16, 17]. Several tools are used to objectively evaluate and diagnose GERD after bariatric surgeries. These include esophagogastroduodenoscopy (EGD), pH studies, high-resolution manometry (HRM), and the Endoluminal Functional Lumen Imaging Probe (EndoFLIP) [18–22]. However, there is no standardized approach for the assessment of GERD in these patients. In this review, we delineate the relationship between GERD and both SG and RYGB, with a focus on the pathophysiology of GERD after bariatric surgeries, its objective assessment on EGD and pH studies, and the underlying anatomical and motility disturbances. We suggest a stepwise algorithm to establish GERD diagnosis after SG and RYGB, determine the underlying cause, and guide management. This algorithmic approach should be considered for patients who have failed maximal medical therapy.

## SG and GERD

SG is the most commonly performed bariatric surgery worldwide. While its association with GERD has been documented, a proportion of patients experience improvement in pre-existing GERD after the surgery [23, 24]. There is a multitude of underlying mechanisms of GERD improvement in these patients, but these are mainly related to the decreased intra-abdominal pressure secondary to weight loss, and the decreased acid production due to the resection of the fundus. Other factors include a decrease in stomach volume and an acceleration of gastric emptying [25, 26].

However, several mechanisms contribute to the development of de-novo GERD or worsening of pre-existing GERD in patients after SG. First, the newly created long and narrow tubular-shaped stomach has decreased compliance, which results in increased intraluminal pressure. Second, several components of the anti-reflux barrier, which consists of the LES, crural diaphragm, and gastroesophageal flap valve, may be damaged during the procedure. The sling fibers of the distal part of the LES can be accidentally transected and the angle of His disrupted, resulting in decreased LES pressure, predisposing patients to reflux. Additional technical factors contributing to GERD include sleeve stenosis or twisting, preserved gastric fundus, and a missed hiatal hernia not repaired at the index procedure [25–29].

The association between SG and GERD has been well established in the literature, yet data have shown variability in the prevalence of subjectively reported post-operative GERD [30]. Most studies have found relatively high rates of GERD after SG, and a recent meta-analysis that included ∼10,000 patients from 46 studies reported worsening of pre-existing GERD in 19% and development of de-novo GERD in 23% of patients [15]. These symptoms persist long-term following surgery, and a prospective multicenter providing >10 years of follow-up after SG reported a significant increase in GERD symptoms from 26.3% before surgery to 58.9% [31].

Endoscopic evidence of both erosive esophagitis (EE) and Barrett’s esophagus (BE) after SG has also been well described. A large cross-sectional study that included 517 patients reported an EE rate of 37.9% after SG, of which 10.7% was severe [32]. Additionally, a recent meta-analysis found BE in 11.6% of patients, with most cases observed 3 years after SG [33]. A prospective multicenter study of SG patients followed for >10 years demonstrated an EE rate of 74.7% and a BE rate of 16.8% [31]. Another meta-analysis reported the presence of EE in 28% and BE in 8% of patients on long-term follow-up after SG [15].

Several studies objectively evaluated GERD with pH studies, and the majority reported an increase in esophageal acid exposure time (EAET) after SG [34–38]. Non-acid reflux is also detected after SG [20, 30, 35]. A meta-analysis reported an increase in the total number of acid and non-acid reflux episodes after SG, along with an increase in both total and recumbent EAET [20].

Anatomical abnormalities, such as hiatal hernia, are commonly observed after SG. Sleeve dilation or stenosis are also reported. One study reported a significant increase in the prevalence of hiatal hernia from 6.1% to 27.3% at 1 year after SG, correlating with the presence of esophagitis [39]. In addition, 12.1% of patients developed a dilated sleeve post-operatively and 6.1% developed stricture at the incisura [39]. All these patients had esophagitis ± GERD symptoms. Another study reported a de-novo hiatal hernia rate of 46% at 1 year after SG, and 11% of the cohort had a hiatal hernia and EE at follow-up evaluation. Almost all (97%) hernias were ≤4 cm in size [40]. In one study that included SG patients with no preoperative reflux or hiatal hernia and who had >10 years of follow-up after surgery, 39% of the cohort showed de-novo hiatal hernia and 14% had BE [41]. In this series, 57% of patients had symptomatic reflux, and these patients were significantly more likely to have a hiatal hernia compared with those without symptoms (52% vs 21%, respectively). Also, sleeve enlargement was found in 57% of patients. Another long-term study reported 58.5% de-novo GERD, 30.2% de-novo esophagitis, and 18.9% de-novo hiatal hernia rates after 10.5 years of follow-up [42].

**Figure 1.**
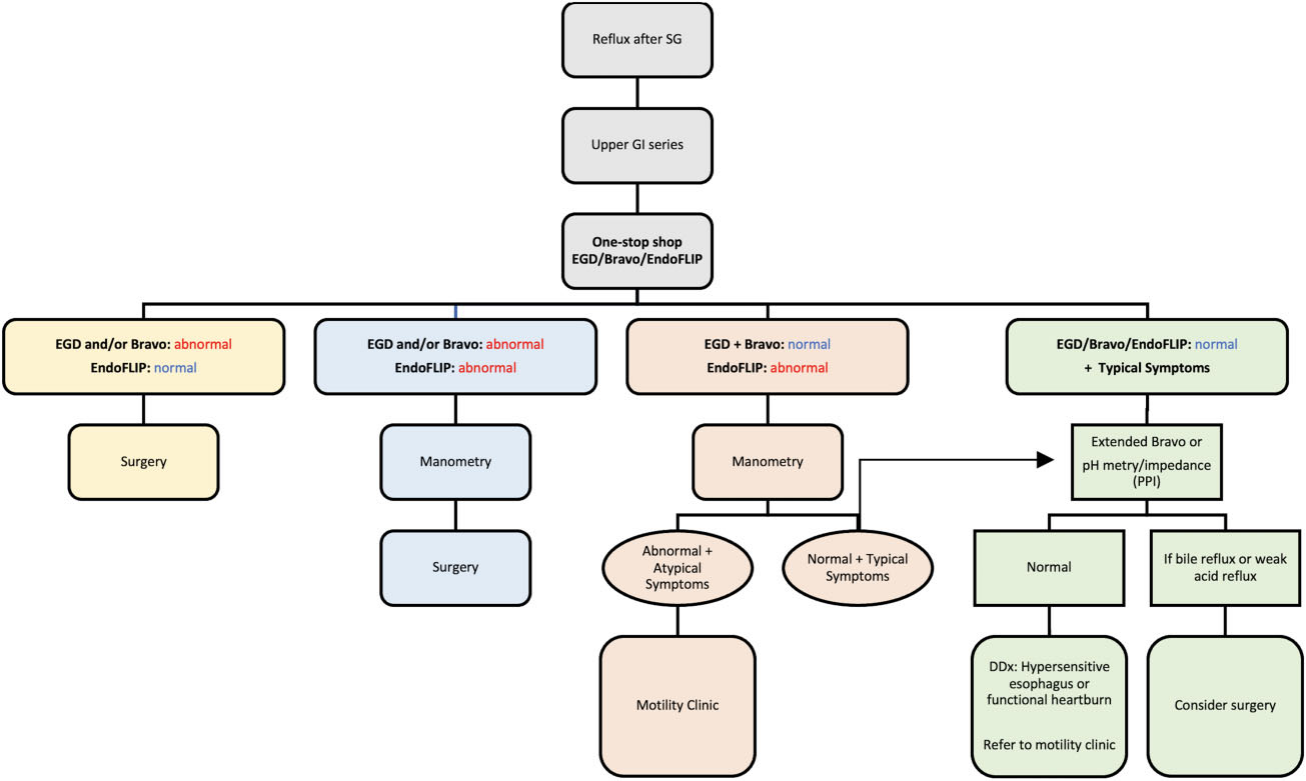
Algorithmic approach for the evaluation and management of GERD after SG. GERD, gastroesophageal reflux disease; SG, sleeve gastrectomy; GI, gastrointestinal; EGD, esophagogastroduodenoscopy; PPI, proton-pump inhibitor; DDx, differential diagnosis.

The effects of SG on esophageal motility have varied in reports and are heterogeneous. While some studies showed a decrease in LES pressure [34, 36, 43, 44], others reported an increase [45] or did not demonstrate a change [23, 35] in LES pressure. Despite this variability, a recent meta-analysis of 612 SG patients reported an overall decrease in both LES pressure and esophageal body amplitude following SG, with an increased risk of ineffective esophageal motility [20]. Another cross-sectional study reported a decrease in both LES pressure and the distal contractile integral after SG [32]. EndoFLIP evaluations of SG patients at 5 years of follow-up showed an increased rate of repetitive retrograde contraction, instead of normal repetitive antegrade contraction [46]. This may relate to increased intragastric pressure, and ensuing increased afterload at the gastroesophageal junction. This entity has been described as post-obesity surgery esophageal dysfunction (POSED) and has been reported in 2.5% of patients after SG [47]. Given the recent description, it is possible that POSED is under-diagnosed and should therefore be considered in the evaluation of GERD in this patient population. We propose an algorithm for the evaluation of post-SG reflux and suggest management options accordingly (Figure 1).

## Evaluation of GERD in patients with SG: algorithm and rationale

### Step 1: Upper gastrointestinal series

Upper gastrointestinal (GI) series is a non-invasive test that may reveal anatomical causes of reflux such as a large hiatal hernia, an enlarged sleeve, an angulated sleeve, or incisural stenosis.

### Step 2: Turnkey evaluation

The Turnkey evaluation includes:

- EGD to assess the presence of esophagitis, determine the Hill grade of the gastroesophageal junction flap valve, and identify the underlying cause of reflux such as enlarged sleeve, sleeve stenosis or twisting, or hiatal hernia.
- Bravo pH study to detect abnormal esophageal acid exposure.
- EndoFLIP to define the motility pattern of the esophagus. A normal EndoFLIP is sufficient to rule out motility disorders [48]. A normal EndoFLIP performed at the time of EGD, therefore, obviates the need for an HRM. An abnormal EndoFLIP should be followed by HRM evaluation to confirm the diagnosis and determine the underlying etiology.

Of note, the initial upper GI series evaluation will further refine the components of the “turnkey evaluation.” For example, a mid-body SG stenosis on the upper GI will ready the endoscopist to provide dilation during EGD without the need for a Bravo pH or EndoFLIP.

#### Abnormal EGD and/or Bravo pH study + normal EndoFLIP

If conclusive evidence is discerned from the Bravo pH study and/or EGD despite an adequate therapeutic trial of proton-pump inhibitors (PPIs) and in the setting of normal EndoFLIP, surgery should be considered to address the underlying anatomical abnormalities. GERD is a common reason for surgical conversion of SG to RYGB, cited as the indication in 30.4% of patients in a meta-analysis [49]. Studies reported an improvement in reflux symptoms after conversion in 70%–100% of patients [50–54].

#### Abnormal EGD and/or Bravo pH study + abnormal EndoFLIP

In this scenario, HRM is the next best step before surgery. When surgery is conducted to alleviate an anatomical abnormality in the presence of established dysmotility, setting expectations with the patient is important as GERD resolution may not always occur.

#### Normal EGD + normal Bravo pH study + abnormal EndoFLIP

We recommend obtaining a manometry to assess for an underlying esophageal motility disorder.

- Abnormal manometry: we recommend referring these patients to the esophageal motility clinic for further evaluation of motility disorders as an underlying cause of reflux.
- Normal manometry: if typical reflux symptoms (heartburn, acid regurgitation) are present, then an extended duration Bravo study (e.g. 72 or 96 h) or a pH impedance while on a PPI is recommended (see below).

#### Normal EGD + normal Bravo pH study + normal EndoFLIP

In the context of typical reflux symptoms despite normal endoscopic, manometric, and pH evaluation, we recommend obtaining an extended duration Bravo pH study (e.g. 72 or 96 h) to increase the diagnostic sensitivity of the test, or a pH impedance test while on a PPI to evaluate for non-acid reflux.

##### Normal extended Bravo pH study or pH impedance test

Disorders of the gut–brain interaction (DGBIs) should be considered, and patients should be referred accordingly to gastroenterology.

##### Abnormal extended Bravo pH study or pH impedance test

Surgery should be considered in this patient group.

## RYGB and GERD

RYGB is the second most performed bariatric surgery after SG. It is commonly performed for the treatment of GERD in patients with obesity, as it has been shown to reduce GERD-related symptoms and the use of heartburn medication, as well as EAET and EE [12, 55–59]. Many factors contribute to the mechanism of GERD improvement after RYGB and include a decrease in intragastric pressure secondary to weight loss, a decrease in acid production due to the exclusion of the fundus, the creation of a lower-pressure gastric pouch isolated from the pylorus, and a decrease in duodenal gastric reflux due to the absence of direct communication between the duodenum and stomach [60–63].

However, some patients experience persisting or worsening pre-existing GERD, and some may develop de-novo GERD after RYGB [12–14, 64]. A randomized clinical trial comparing RYGB with SG included 110 patients with RYGB and reported de-novo reflux symptoms in 10.7% and worsening of reflux in 6.3% of patients at 5 years after RYGB, while 60.4% experienced remission of GERD [13]. De-novo GERD after RYGB was reported to occur in 2.3% and 7.3% of patients in two studies [14, 64]. The underlying mechanisms of GERD after RYGB are mainly related to the presence of a hiatal hernia, a large or enlarged gastric pouch, damage to the sling fibers during dissection, esophageal dysmotility, development of a gastrogastric (GG) fistula, and bile reflux due to a short Roux limb [59, 61, 62].

A cross-sectional study of 256 RYGB patients reported an EE rate of 17.6% and a BE rate of 5% at a median of 8 years after surgery [32]. A recent prospective trial that included only patients with >10 years of follow-up reported an EE rate of 22.0% after RYGB, without cases of BE [31]. Despite the significant rate of EE described in these studies, both reported a significantly higher rate of EE after SG compared with RYGB.

In terms of acid exposure, most studies report a decrease in EAET after RYGB. However, non-acid reflux seems to play a role in the pathophysiology of GERD in these patients. In one study, 31% of patients with typical GERD symptoms were found to have a positive symptom index (SI) for non-acid reflux (NAR), and 19% of patients with atypical symptoms had a positive SI for NAR [65]. In another study, 38% of patients with GERD symptoms had general reflux, defined as a positive SI for major, minor, and NAR combined, but not for major acid reflux alone [66]. In a meta-analysis, an increased number of NAR events was observed, despite decreased acid reflux and unchanged total reflux episodes [20].

The impact of RYGB on esophageal motility remains unclear, with studies reporting a decrease in LES pressure [67, 68] while others found no changes [69, 70]. Similarly, esophageal body contraction was decreased in some studies [55, 71], and unchanged [69, 70] or improved [55] in others. Overall, a recent systematic review and meta-analysis of 470 RYGB patients reported an increased risk of ineffective esophageal motility but an unchanged LES pressure [20]. Additionally, POSED has also been described after RYGB, with a reported prevalence of 6.9% [47].

Patients with RYGB and reflux require a comprehensive evaluation to objectively assess GERD and determine its underlying etiology. One study evaluated patients with GERD after RYGB using barium swallow, esophagogastroduodenoscopy (EGD), and 24-h pH impedance manometry [72]. Most patients presented with typical GERD symptoms (93.6%). At a median of 3.8 years of follow-up, 53.2% of patients had a hiatal hernia, 10.6% had an enlarged pouch, and 5.1% had a GG fistula. Esophagitis Los Angeles grade of >B was observed in 23.4% of patients. Abnormal motility was observed in a significant portion of patients, including esophageal hypomotility or aperistalsis in 37.8% of patients, and a hypotensive LES in 57.8% of patients. Also, pH studies showed an increased EAET in 61.4% of patients and an increased number of reflux episodes in 68.2%. Interestingly, 12.8% of patients were diagnosed with a functional disorder, defined as GERD symptoms in the absence of inflammatory, structural, or major motility disorders.

**Figure 2.**
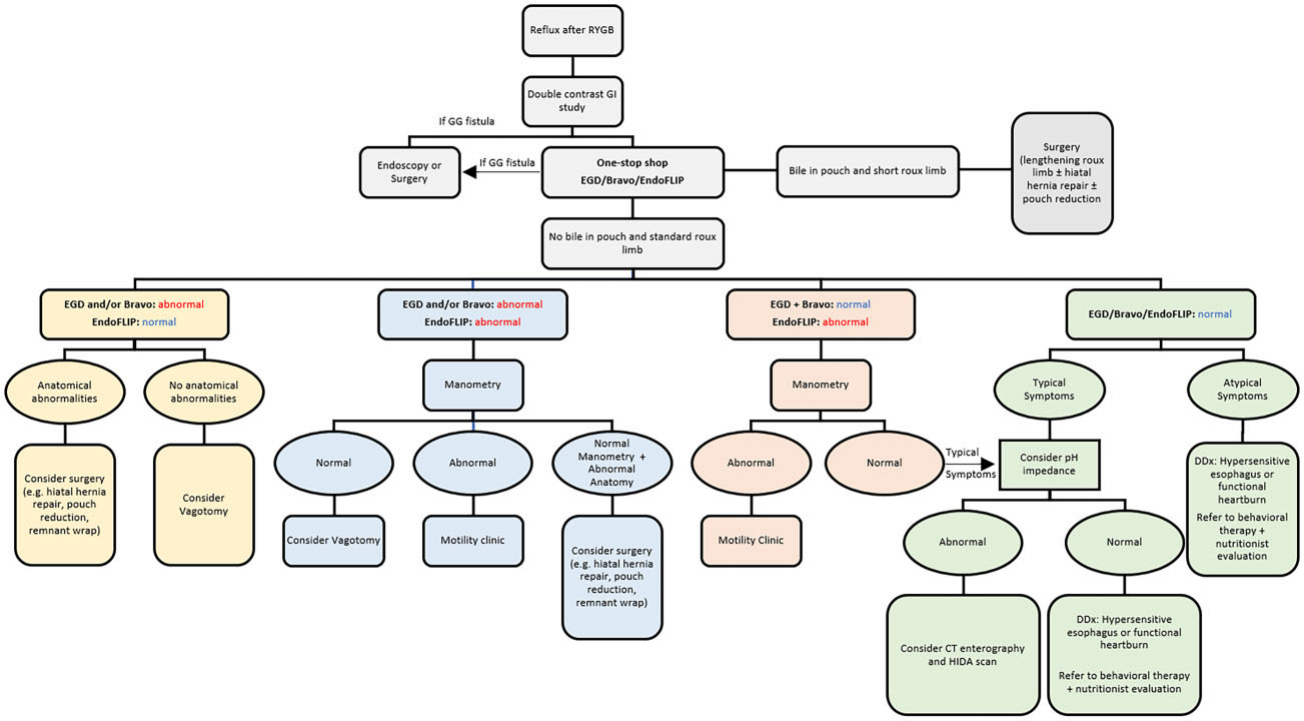
Algorithmic approach for the evaluation and management of GERD after RYGB. GERD, gastroesophageal reflux disease; RYGB, Roux-en-Y gastric bypass; GI, gastrointestinal; GG, gastrogastric; EGD, esophagogastroduodenoscopy; CT, computed tomography; HIDA, hepatobiliary iminodiacetic acid; DDx, differential diagnosis.

Our algorithm for evaluating and managing patients with post-operative GERD after RYGB (Figure 2) is detailed below.

## Evaluation of reflux in patients with RYGB: rationale and algorithm

Several structural abnormalities in the context of RYGB may provoke the occurrence of GERD in these patients. These include the presence of a distended pouch, an anastomotic stricture at the gastrojejunostomy or downstream at the jejunojejunostomy, the presence of a GG fistula transmitting the pressure from the excluded stomach, and the presence of a hiatal hernia. We discuss possible approaches to investigate GERD in RYGB patients with these abnormalities in mind [73–75].

### Step 1: Double-contrast upper GI series

Double-contrast upper GI series is a non-invasive test that would help outline structural abnormalities. If the study is positive, an EGD can be done for risk stratification and further therapy. A subcentimeter GG fistula can be successfully treated endoscopically with clips, stents, or sutures, with a reported high success rate of 76% with endoscopic stenting [76, 77]. However, a GG fistula of size >1 cm are less likely to heal endoscopically [77], and surgical revision may eventually be necessary.

### Step 2: Turnkey evaluation

This includes EGD with ensuing Bravo pH and/or EndoFLIP in the same session (see above). As noted above, EGD allows risk stratification. For example, if bile is seen in the pouch, this suggests a GG fistula or a short Roux limb. Bile in contact with gastric mucosa can be ulcerogenic and results in gastritis [78]. Although few studies have assessed the incidence of bile reflux after RYGB, rates of 3.4%–5% have been reported in the literature [79, 80]. These patients may benefit from Roux-limb lengthening [78]. Other concomitant structural abnormalities, such as the presence of a hiatal hernia or dilated gastric pouch, can be addressed surgically or endoscopically.

#### Abnormal EGD and/or Bravo pH study + normal EndoFLIP

A normal EndoFLIP obviates the need for HRM.

- Surgery or interventional endoscopy should be considered to address anatomical abnormalities such as hiatal hernia or enlarged gastric pouch.
- In the setting of normal anatomy and normal EndoFLIP findings, an abnormal EAET on Bravo pH study is likely secondary to increased acid production in the pouch. If acid production is not responsive to acid suppression, vagotomy has been described to decrease acid production in the stomach [81]. Selective vagotomy and highly selective vagotomy have a limited role in the setting of RYGB and truncal vagotomy is preferred. Thoracoscopic truncal vagotomy (TTV) has been assessed for the treatment of refractory marginal ulcers in RYGB patients. One study comparing TTV to surgical revision of the gastrojejunal anastomosis reported similar safety and efficacy between the two procedures, with only 14% having recurrence after TTV [82].

#### Abnormal EGD and/or Bravo pH study + abnormal EndoFLIP

In this scenario, a manometry is recommended to assess for an underlying esophageal motility disorder.

- Normal manometry: in the setting of normal anatomy, the increased EAET on Bravo pH is attributed to increased acid production. If refractory to acid suppression, vagotomy can be considered.
- Normal manometry + anatomical abnormalities: surgery should be considered (e.g. hiatal hernia repair ± surgical pouch reduction).
- Abnormal manometry: we recommend referring these patients to the esophageal motility clinic for further evaluation of motility disorders as an underlying cause of reflux.

#### Normal EGD + normal Bravo pH study + abnormal EndoFLIP

We recommend obtaining a manometry to assess for an underlying esophageal motility disorder.

- Abnormal manometry: these patients should be referred to the esophageal motility clinic for further evaluation.
- Normal manometry: in the setting of normal EGD and Bravo pH study and typical reflux symptoms, we recommend obtaining a pH impedance test while on a PPI to assess for NAR (see below).

#### Normal EGD + normal Bravo pH study + normal EndoFLIP

In the context of typical reflux symptoms despite normal EGD, Bravo pH study, and EndoFLIP findings, we recommend obtaining a pH impedance test while on a PPI to evaluate for NAR.

- Abnormal pH impedance test: we recommend obtaining a computed tomography (CT) enterography to evaluate for occult GG fistula and to estimate the length of the Roux limb, and an extended hepatobiliary iminodiacetic acid scan to evaluate for bile reflux into the pouch or esophagus.
- Normal pH impedance test: DGBI should be considered, and patients referred accordingly to gastroenterology.

## Other bariatric surgeries

Although other bariatric surgeries exist, the discussed approach can still be universal. Patients with duodenal switch (DS) can be managed in the same way as those who have received SG. However, the conversion of DS to RYGB is technically complex due to pre-existing surgical anastomoses. DS patients with refractory GERD, and in whom no surgically correctable anatomic abnormality is discerned, may thus be best managed with endoscopic anti-reflux therapies. For patients with GERD and a gastric band (a procedure that has been gradually abandoned), readjusting the band tightness may be key to alleviating GERD. In this patient population, esophageal dysfunction may occur, with mega-esophagus in extreme cases, especially if band tightening is not addressed early, and behavioral modifications for meal size and consumption are not emphasized.

## Conclusions

In this review, we have discussed a stepwise approach for the evaluation of GERD after SG and RYGB, to help guide physician decision-making. We recommend an upper GI series as an initial non-invasive test that would help identify obvious anatomical abnormalities. Next, we recommend a “turnkey evaluation” consisting of an EGD, Bravo pH study, and EndoFLIP, to objectively assess GERD and determine the underlying anatomical or motility etiology. A normal EndoFLIP at the time of EGD obviates the need for HRM. HRM can be requested to confirm abnormal EndoFLIP findings and determine the etiology. In case of negative EGD, pH, and motility testing, a pH impedance test would help detect abnormal non-acid esophageal exposure as a possible cause of reflux. An extended duration Bravo pH study can also be considered to increase the diagnostic sensitivity of the test. Subsequent management depends on the test findings and can include hiatal hernia repair, conversion to a RYGB, vagotomy, reduction of the gastric pouch size, lengthening of the Roux limb, or other surgical or endoscopic interventions, as well as referral to the GI motility clinic for further evaluation. Patients with negative evaluations may suffer from DGBI and should be referred to behavioral therapy, nutritional counseling, and gastroenterology.

## Authors’ Contributions

O.M.G., B.K.A.D., and R.G. conceived and design the manuscript; O.M.G., B.K.A.D., R.G., F.A.R., and F.B. drafted the manuscript; O.M.G., B.K.A.D., R.G., F.A.R., F.B., K.R., L.K., and S.N.K. critically revised the manuscript. All authors have read and approved the final version of the manuscript.
